# Mutation of arginine residues to avoid non-specific cellular uptakes for hepatitis B virus core particles

**DOI:** 10.1186/s12951-015-0074-8

**Published:** 2015-02-13

**Authors:** Izzat Fahimuddin Bin Mohamed Suffian, Yuya Nishimura, Kenta Morita, Sachiko Nakamura-Tsuruta, Khuloud T Al-Jamal, Jun Ishii, Chiaki Ogino, Akihiko Kondo

**Affiliations:** Department of Chemical Science and Engineering, Graduate School of Engineering, Kobe University, Kobe, Japan; Organization of Advanced Science and Technology, Kobe University, Kobe, Japan; Institute of Pharmaceutical Science, Faculty of Life Sciences and Medicine, King’s College London, London, UK

## Abstract

**Background:**

The hepatitis B virus core (HBc) particle is known as a promising new carrier for the delivery of drugs and nucleic acids. However, since the arginine-rich domain that is located in the C-terminal region of the HBc monomer binds to the heparan sulphate proteoglycan on the cell surface due to its positive charge, HBc particles are introduced non-specifically into a wide range of cells. To avoid non-specific cellular uptake with the intent to control the ability of cell targeting, we individually replaced the respective arginine (R) residues of the arginine-rich domain located in amino acid positions 150–159 in glycine (G) residues.

**Results:**

The mutated HBc particles in which R154 was replaced with glycine (G) residue (R154G) showed a drastic decrease in the ability to bind to the heparan sulphate proteoglycan and to avoid non-specific cellular uptake by several types of cancer cells.

**Conclusions:**

Because this mutant particle retains most of its C-terminal arginine-rich residues, it would be useful in the targeting of specificity-altered HBc particles in the delivery of nucleic acids.

**Electronic supplementary material:**

The online version of this article (doi:10.1186/s12951-015-0074-8) contains supplementary material, which is available to authorized users.

## Background

Hepatitis B virus core (HBc) particles have been studied as promising virus-like particles (VLPs) to serve as carriers in drug delivery systems (DDSs) [1,2]. HBc particles consist of 180 (T = 3) or 240 (T = 4) units of HBc monomers that have the ability to form an icosahedral capsid [3,4]. Coordinating salt and urea concentrations enable control of the phases between assembly and disassembly of the HBc capsid [5]. HBc monomers are composed of two distinct domains: i) an assembly domain (amino acid residues (aa) 1–149) that drives particle formation, and ii) an arginine-rich domain (aa 150–183) that recognizes the cell surface heparan sulphate proteoglycan with an electrostatic interaction [6]. The heparan sulphate proteoglycan is known as a major physiological ligand for many heparin-binding proteins [7]. Additionally, the arginine-rich domain behaves as a binding site for nucleic acids, because of its positively charged residues [8,9].

It has been demonstrated that the engineered HBc monomer deleting the entire arginine-rich domain (aa 150–183) could associate and form a particle structure but it could not bind the cells [10,11]. In particular, the aa 150–162 of HBc is necessary, whereas the aa 163–183 is dispensable for heparan sulphate proteoglycan-mediated cell attachment, even though the aa 160–183 is useful as the binding site to nucleic acid medicine [12]. Thus, there is no doubt that the arginine residues in aa 150–159 serve the cell binding and the uptake. However, the question remains as to which of the aa 150–159 in the arginine-rich domain will bind to the heparan sulphate proteoglycan. To employ HBc particles for the targeted cell-specific delivery of nucleic acids, it is important to understand the arginine residues involved in the non-specific cellular uptake of HBc particles.

In this research, we performed site-directed mutagenesis for the HBc monomer to identify the amino acid residues concerned in the binding to the heparan sulphate proteoglycan. Each arginine (R) residue among aa 150–159 of the arginine-rich domain in the HBc monomer was individually replaced with a glycine (G) residue, and the cellular uptakes of the mutated HBc particles were evaluated. The HBc particle introducing the R154G mutation showed a drastic decrease in all capacities of cellular uptake for HeLa, NuE and A431 cells. Our results would be useful in the engineering of HBc particles to serve as carriers with cell-specific targeting for nucleic acid delivery.

## Results and discussion

**Figure 1:**
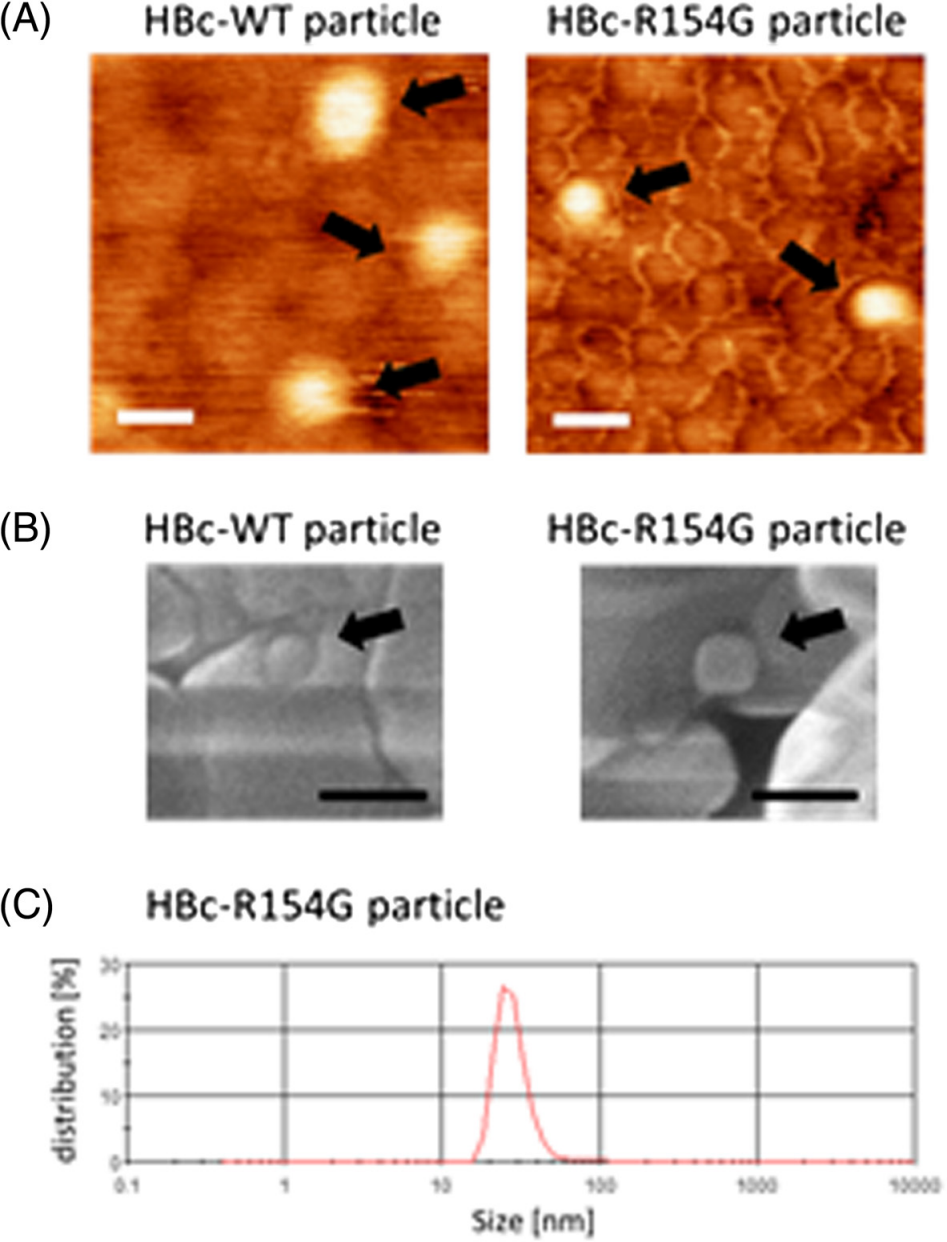
**Analyses of purified HBc particles. (A)** Atomic force microscope images of HBc-WT particle (left) and HBc-R154G particle (right). Scale bar: 50 nm. **(B)** Scanning electron microscope images of HBc-WT particle (left) and HBc-R154G particle (right). Scale bar: 100 nm. **(C)** Size distribution using DLS analysis. The average size of the HBc-R154G particle was 28.7 nm.

Wild-type and singly mutated (respectively replacing R with G among aa 150–159 in the arginine-rich domain) HBc monomers (Additional file 1) were expressed in *E. coli*, and the proteins were extracted with lysis buffer as well as with dissociation buffer. HBc dimers were then purified by affinity chromatography. It has been proved that the C-terminal histidine-tag on HBc monomer had no significant adverse effect on the particle formation and the cell binding [11]. The expression of each HBc monomer (21 kDa) was confirmed by western blot analysis using anti-His6 antibody (data not shown). The particle formation was confirmed by atomic force microscopy (AFM), scanning electron microscope (SEM) and dynamic light scattering (DLS) (Figure 1). These results indicated that point-mutations replacing R with G in the arginine-rich domain (150–159 aa) did not affect the self-assembly capacity of the HBc dimers.

**Figure 2:**
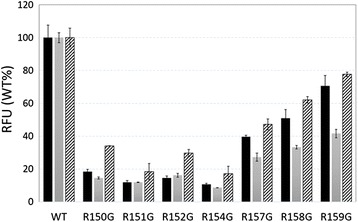
**Relative fluorescence units (RFU) of HeLa, NuE and A431 cells treated with Alexa Fluor 488-labeled HBc particles.** (Final concentration of Alexa Fluor 488-labeled HBc particles: 10 μg/ml) Black bars, HeLa cells; grey bars, NuE cells; and, striped bars, A431 cells.

To evaluate the cell-binding capability of singly mutated HBc particles, each HBc particle was labelled with Alexa Fluor 488. HeLa, A431 and NuE cells were then treated with the labelled HBc particles. After washing the cells to remove the non-bound HBc, the green fluorescence of the cells was analyzed using a flow cytometer. The fluorescence intensity of the cells treated with wild-type HBc particles was measured in relative fluorescence units (RFUs) (Figure 2). Although the relative ability of all singly mutated HBc particles to bind with NuE cells was lower than the ability to bind to HeLa and A431 cells, the relative binding of wild-type HBc and the mutants was consistent among different cell types. The replacement of the R residues at aa 157, 158 and 159 (R157G, R158G and R159G) showed a comparatively higher cell binding ability compared with other HBc mutants. The mutations of R residues at aa 150, 151, 152 and 154 (R150G, R151G, R152G and R154G) in HBc considerably decreased the cell binding ability. Among them, the R154G mutation of HBc was the most effective in decreasing the cell binding ability to all three cell types, while its potency was fairly close to those of R150G, R151G and R152G. Thus, the R154 residue and its peripheral R residues (aa 150–152) in the arginine-rich domain are critical to the cell binding ability of HBc particles, and the HBc-R154G particles would be useful in the development of an engineered HBc particles for the targeted cell-specific delivery of nucleic acids.

**Figure 3:**
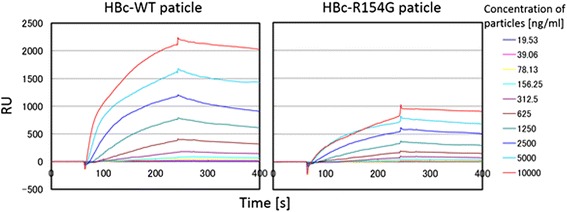
**The concentration-dependent binding curves of HBc-WT and mutated HBc particles.** The interaction with heparan sulfate proteoglycan was analyzed by Biacore.

Proteins possessing either an arginine-rich domain or a protein transduction domain (PTD) will bind to the heparan sulfate proteoglycan on a cell surface [13,14]. To examine the binding affinity of mutated HBc particles with a heparan sulfate proteoglycan, we performed surface plasmon resonance (SPR) analysis (Figure 3). The binding curve of the HBc-R154G particle was lower than that of wild-type HBc particles, which agreed with the results found using a flow cytometer. The value of *k*_*a*_ showed a 1.5-fold difference between WT-HBc (1.45 × 10^7^) and HBc-R154G (9.44 × 10^6^) particles. These results indicated that the arginine residue at aa 154 is surely involved in binding to the cell surface of a heparan sulfate proteoglycan.

**Figure 4:**
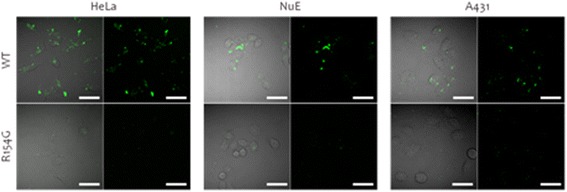
**Fluorescence images of HeLa, NuE and A431 cells treated with Alexa Fluor 488-labeled HBc particles.** (Final concentration of Alexa Fluor 488-labeled HBc particles: 10 μg/ml) The cells were observed using a confocal laser-scanning microscope: Scale bars, 50 μm.

To evaluate the effect of R154G mutation on cellular uptake, HeLa, NuE and A431 cells were treated with HBc-WT and HBc-R154G particles labelled with Alexa Fluor 488. After incubation for 3 h, the cells were observed by confocal laser-scanning microscope (CLSM) (Figure 4). Green fluorescent signals of HBc-WT particles were observed clearly in all three cell types. In contrast, the green fluorescence of HBc-R154G particles was little observed in any three cell types. This result indicated that the HBc-R154G particles showed the decrease of the non-specific cellular uptake ability for three different cell types.

## Conclusions

Wild-type HBc particles have the ability to bind to a wide range of cells due to the C-terminal arginine-rich domain that interacts with the cell surface of a heparan sulfate proteoglycan. Arginine residues located within aa 150–159 among the arginine-rich domain were thought to be related to the interaction [12]. Therefore, singly mutated HBc particles in which each arginine residue (aa 150–159) was replaced with glycine residues were prepared. As a result, the cell-binding abilities of most of the mutated HBc particles were decreased compared with wild-type HBc particles. In particular, the HBc-R154G particles displayed the lowest degree of cell binding ability. The HBc-R154G particles showed a clear decrease in the binding ability to a heparan sulfate proteoglycan, as well as a decrease in the cellular uptake capacity. Therefore, the replacement of an arginine residue at the aa 154 position was critical to avoid non-specific cellular binding and uptake. Thus, the R154-mutated HBc particles would be useful in the development of specificity-altered HBc for targeted nucleic acid delivery.

## Methods

### *Plasmid construction of wild-type and singly mutated HBc*

The plasmid pET-22b-HBc [15] was used to prepare a wild-type HBc particle containing a histidine-tag (His6) at the C-terminus (HBc-WT-His6). To prepare HBc particles with a single mutation (HBc-R15XG-His6, X = 0, 1, 2, 4, 7, 8, 9), each arginine residue was replaced with glycine residue in plasmids expressing singly mutated HBc monomers that were constructed as follows. DNA fragments encoding HBc-R15XG-His6 (X = 0, 1, 2, 4, 7, 8, 9) were amplified by polymerase chain reaction (PCR) from pET-22b-HBc with the the following primers: (5′- TAA TCT CGA GTC TAG AGA ATT AGT AGT CAG CTA TGT -3′ and 5′- CCC CCG CGG CGA GGG AGT TCT TCT TCT AGG GGA CCT GCC TCG TCG TCT AAC AAC AGT AGT TTC -3′ replacing each R with G) based on Additional file 1. The amplified fragments and pET-22b-HBc were digested with *Xba*I/*Sac*II, and were ligated at the same sites. The resultant plasmids were designated as pET-22b-HBc-R15XG-His6 (X = 0, 1, 2, 4, 7, 8, 9).

### *Expression of HBc monomers inEscherichia coli*

Each plasmid expressing wild-type and singly mutated HBc monomers was introduced into *Escherichia coli* BL21 (DE3). The cultures of the transformants (4 ml) were inoculated into 1 L of fresh LB-media (1% tryptone, 0.5% yeast extract, 0.5% NaCl) containing 100 μg/ml ampicillin and grown at 37°C with shaking at 150 rpm until the OD_600_ reached 0.7 ~ 0.8. Then, protein production was induced by adding isopropyl-β-thiogalactopyranoside (IPTG) with a final concentration of 100 μM at 25°C overnight. Cells were collected at 3,000 rpm for 15 min, and the sediment was used for purification.

#### Purification of HBc particles

Each HBc particle was purified as reported previously [16]. Briefly, a cell pellet was suspended in 30 ml of lysis buffer (pH 8.0) (50 mM Tris–HCl, 100 mM NaCl, 5 mM EDTA, 0.2% Triton X-100, 10 mM β-mercaptoethanol, 10 mg/ml DNAse I, 10 mg/ml RNAse A) with a vortex. The cells were lysed on ice by 3 cycles of sonication for 1 min each at 1 min intervals to avoid heating of the material. The supernatant was removed by centrifugation at 15,000 rpm and 4°C for 30 min. The HBc particles in the pellet were twice washed in 50 ml of lysis buffer and each time collected by centrifugation at 12,000 rpm and 4°C for 15 min. The HBc particles and contaminating *E. coli* proteins were dissolved in 25 ml of dissociation buffer (pH 9.5) (4 M urea, 200 mM NaCl, 50 mM sodium carbonate, 10 mM β-mercaptoethanol) by overnight incubation in a refrigerator at 4°C. After the addition of 10 ml of dissociation buffer, the preparation was incubated for an additional 2 h on ice.

Contaminating proteins were separated from HBc proteins using denaturing affinity chromatography. A column with 10 ml of Ni-agarose (COSMOGEL His-Accept; Nacalai Tesque, Kyoto, Japan) was equilibrated with 5 ml of dissociation buffer in 3 cycles. The preparation was loaded onto the equilibrated column and washed with 5 ml of dissociation buffer in 3 cycles. Bound proteins were eluted with 10 ml of elution buffer (pH 9.5) (dissociation buffer containing 1 M imidazole), and the elution was collected into 1 ml fractions. Each fraction was separated by 15% sodium dodecyl sulphate-polyacrylamide gel electrophoresis (SDS-PAGE), and stained with Coomassie brilliant blue (CBB) to analyse its purity. Fractions containing the pure proteins were polymerized to HBc particles by removal of the urea in the dialysis buffer (pH 7.0) (500 mM NaCl, 50 mM Tris–HCl, 0.5 mM EDTA) overnight. Dialysed HBc particles were obtained through a 0.22 μm filter in 3 cycles. The concentration was measured using a Protein Assay Bicinchoninate Kit (BCA Protein Assay) (Nacalai Tesque).

#### Western blotting

The expression of each HBc particle was determined by western blot analysis using a polyvinilidene fluoride (PVDF) membrane. Rabbit anti-6-His antibody (Bethyl Laboratories, Montgomery, TX, USA) was used for the immunoblotting, followed by alkaline phosphatase (AP) conjugated anti-rabbit IgG antibody (Promega, Madison, WI, USA). The membrane was stained with 5-bromo-4chloro-3-indolyl phosphate (BCIP) and nitro blue tetrazolium (NBT) (Promega).

#### Atomic force microscope (AFM)

One hundred microliter solution containing HBc particles was deposited on mica surfaces (11 mm × 11 mm × 0.15 mm) at room temperature for 5 minutes, and then flushed with air. Tapping mode AFM analysis (TM-AFM) was carried out in air at 25°C using a Bruker Dimension ICON with ScanAsyst® (Bruker UK Ltd, Coventry, United Kingdom). The surface was imaged with a tapping tip mode by MikroMasch in Estonia (NSC15/no Al, tip radius < 10 nm; tip height = 20–25 μm; cone angle < 40°, cantilever thickness = 3.5-14.5 μm; cantilever width = 28–32 μm; cantilever length = 120–130 μm; frequency *f*_*0*_ = 265–400 kHz; force constant k = 20–75 N m^−1^, VEECO, USA). The statistical analysis of the AFM images was carried out using WSxM v5.0 Developed 6.2 software (Nanotec Electronica S.L., Madrid, Spain).

#### Scanning electron microscope (SEM)

The freeze-dried HBc particles were analyzed using a JSM-7500 F (JEOL, Munchen, Germany), following the manufacturer’s procedure.

#### Dynamic light scattering (DLS)

The diameter of HBc particles was measured using a Zetasizer Nano ZS (Malvern Instruments, Worcestershire, UK), following the manufacturer’s procedure.

#### Cell culture

HeLa and A431 cells were cultured in Dulbecco’s modified Eagle’s medium (DMEM) (Nacalai Tesque) containing 10% fetal bovine serum (FBS) (Nacalai Tesque), 5% penicillin and streptomycin in the presence of 5% of CO_2_ at 37°C. NuE cells were cultured in RPMI1640 medium (Nacalai Tesque) containing 10% fetal bovine serum (FBS), 5% penicillin and streptomycin in the presence of 5% of CO_2_ at 37°C.

#### Evaluating the cell binding ability of HBc particles

Purified HBc particles were reacted with Alexa Fluor 488 succinimidyl esters (Molecular Probes/Life Technologies, Carlsbad, CA) for 1 h at room temperature under shading. The mixture then was dialyzed with dialysis buffer overnight to remove the free Alexa Fluor 488 [17]. Approximately 1 × 10^5^ units of HeLa, A431 and NuE cells were seeded per well into 12-well plates and cultured overnight. The cells were washed with phosphate-buffered saline (PBS) (Nacalai Tesque) and treated with each particle in serum-free medium at 37°C for 1 h. The final concentrations of core particles were 10 μg/ml for each cell. The cells were then washed twice with serum-free medium and treated with fresh-serum medium at 37°C for 2 h. After washing with PBS, the green-fluorescence was analyzed using a BD FACSCanto II flow cytometer (BD Biosciences, San Jose, CA, USA).

#### Surface Plasmon resonance (SPR) analysis

The interaction between HBc particles and heparan sulfate proteoglycan was measured using a Biacore 3000 (GE Healthcare, Piscataway, NJ, USA) [13]. A sensor chip SA (GE Healthcare) immobilizing heparin sodium salt from porcine intestinal mucosa (Sigma-Aldrich, St. Louis, MO, USA) was prepared using an amine coupling method, according to the manufacturer’s procedure. Each HBc particle was dissolved in running buffer (HBS-EP buffer: 0.01 M HEPES, 0.15 M NaCl, 3 mM EDTA, 0.005% Surfactant P20, pH 7.4) (GE Healthcare) and loaded onto the sensor chip. The chip was regenerated in 1 M NaCl buffer. As the experimental curve-fitting methodology, a 1:1 Langmuir binding model was used. Each HBc particle was dissolved in running buffer and loaded onto the sensor chip. The signal data were collected using Biacore 3000 Control Software.

#### Evaluating the cellular uptake of HBc particles

Approximately 2 × 10^4^ units of HeLa, A431 and NuE cells were seeded in 35 mm glass-based dishes (Iwaki/AGC Techno Glass, Tokyo, Japan). After incubation for 24 h, the cells were washed with PBS and treated either with HBc-WT or HBc-R154G in serum-free medium at 37°C for 1 h. The final concentration of the particles was 10 μg/ml. The cells were then washed twice with serum-free medium and treated with fresh-serum medium at 37°C for 2 h. The cells were observed using a CLSM 5 PASCAL (Carl Zeiss, Oberkochen, Germany) confocal laser-scanning microscope.

## Additional file

Additional file 1:
**Base and amino acid sequences of HBc particles.**
