# Supplementary material for: Mutation of arginine residues to avoid non-specific cellular uptakes for hepatitis B virus core particles
Source: J Nanobiotechnology. 2015 Feb 13;13:15. doi: 10.1186/s12951-015-0074-8 (PMC4334417; doi:10.1186/s12951-015-0074-8)
Supplement: Additional file 1: — Base and amino acid sequences of HBc particles. [file 12951_2015_74_MOESM1_ESM.doc]

Additional file 1 Base and amino acid sequences of HBc particles.


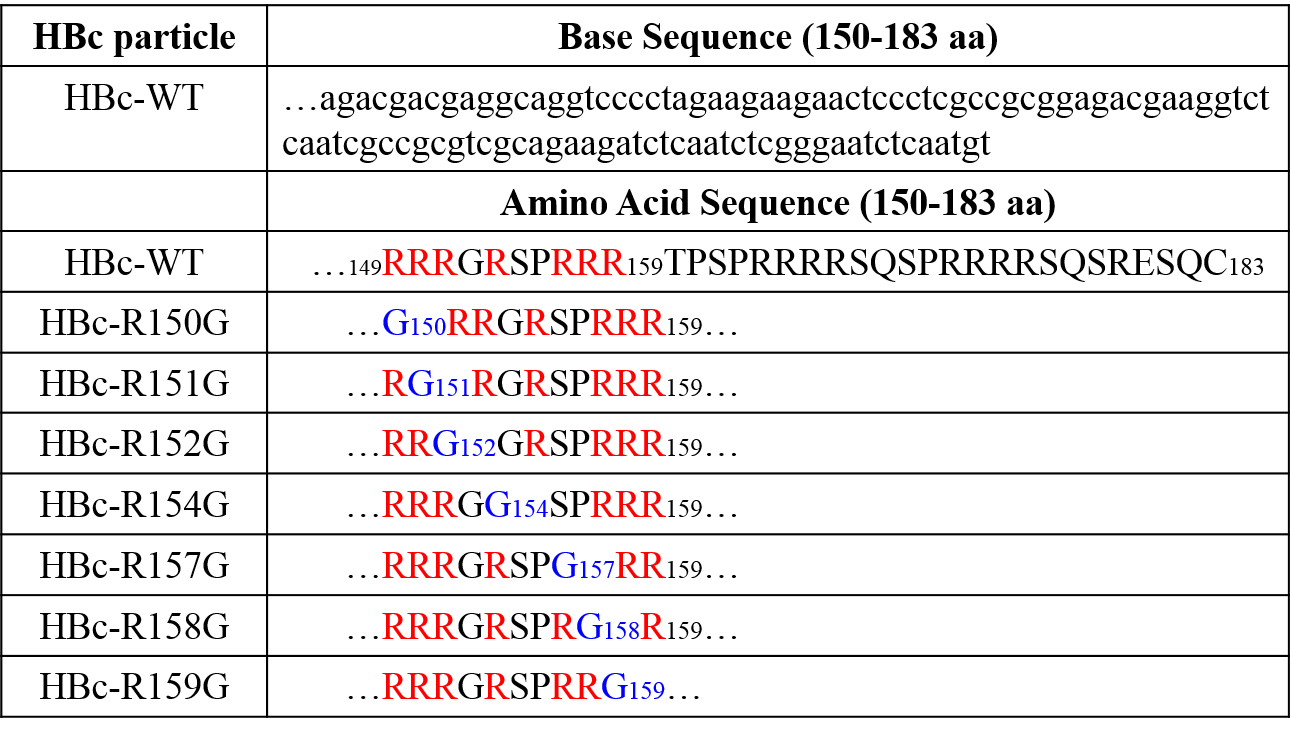


Arginine residues (150-159 aa) (R: red) were replaced with glycine residues (G: blue).
